# Differences Between the Psychological Symptoms of Health Workers and General Community After the First Wave of the COVID-19 Outbreak in Spain

**DOI:** 10.3389/fpsyg.2021.644212

**Published:** 2021-09-03

**Authors:** Sergio Reno-Chanca, Julie Van Hoey, Jesús Alberto Santolaya-Prego de Oliver, Ilargi Blasko-Ochoa, Pilar Sanfeliu Aguilar, Carmen Moret-Tatay

**Affiliations:** ^1^Facultad de Psicología, Universitat Jaume I, Castellón de la Plana, València, Spain; ^2^Kayros Rresearch Group, València, Spain; ^3^Facultad de Psicología, Universidad Europea de Valencia, València, Spain; ^4^Mind, Emotion and Behavioural Research Laboratory (MEB Lab), Universidad Católica de Valencia San Vicente Mártir, València, Spain; ^5^Facultad de Psicología, Universidad CEU Cardenal Herrera, València, Spain; ^6^Dipartimento di Neuroscienze Salute Mentale e Organi di Senso (NESMOS) Università Sapienza di Roma, Rome, Italy

**Keywords:** mental health, COVID-19, distress, OCD, health care workers, psychologist

## Abstract

The coronavirus disease-2019 (COVID-19) has worsened the physical and mental health of the general population. Healthcare workers have a high risk of suffering a mental disorder after the first wave. In this way, psychologists, who deal with mental health issues and are considered as healthcare workers in many countries, are of interest in this context. The present study aimed to examine anxiety, depression, stress, and obsessions and compulsions across psychologists, healthcare professionals, and the general community. These variables were measured through the Depression, Anxiety, and Stress Scale (DASS-21), as well as the Yale–Brown Obsessive Compulsive Scale (Y-BOCS), which are related to different sociodemographic variables. The study was carried out after the first wave in Spain through an online questionnaire. Structural equation modeling and a multigroup analysis were carried out across the groups and variables under study. The results suggested that; (i) healthcare workers and general community depicted similar results in anxiety and stress, as well as obsessions; (ii) the group of psychologists depicted better scores than the other groups under study; (iii) stress and anxiety did not predict compulsions in the group of psychologists; (iv) anxiety predicted obsessions for all the professions, while the relationship of this variable with stress was different for each group; and (v) invariance reached a full metric level.

## Introduction

**Figure 1:**
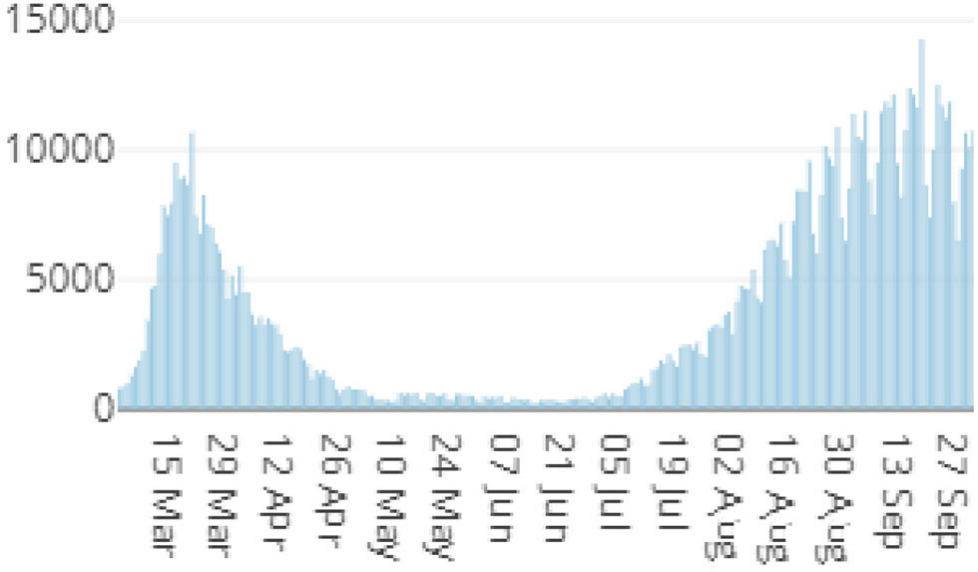
Number of cases reported between the first wave and the second wave in Spain [adapted from the Ministerio de Sanidad (2021); see https://cnecovid.isciii.es/covid19/].

Since the first reported cases related to the coronavirus disease-2019 (COVID-19) in December 2019, humanity is dealing with a large body of preventive actions to contain the spread of this new virus. According to the report on May 15, 2020, by the Spanish Ministry of Health (2020), the situation in Spain was considered alarming, having reported 230,183 cases and 27,459 deaths. At that moment, the restriction measures in Spain, as in other many countries, involved governmental and regional actions, such as travel restrictions, nighttime curfew, and limitations of public or private persons, among others. As depicted in Figure 1, which reflects the cumulative incidence throughout the year, the number of reported cases decreased over time, giving way to the *new normality* in Spain. This new state started in June after the *state of alarm* ended. Nevertheless, it is necessary to consider the low diagnostic capacity during the first wave, in which real incidents were higher.

From June 21, 2020, all the citizens were allowed to move freely across all the regions of Spain. Although the restriction measures became more flexible at that time, the American Psychological Association (2020) emphasized that the habits, personal and professional projects, and forms of social interaction among the population were disturbed. These circumstances, while not necessarily leading to a clinical worsening of the population, may generate a certain level of uncertainty around the psychological consequences (Castiglioni and Gaj, 2020).

The side effects of restriction have been described in several fields, such as the social or economic one (Bonaccorsi et al., 2020). Not surprisingly, there have been concerns regarding the mental health and psychological distress during the COVID-19 outbreak. According to the literature, the effects of mass lockdown have led to feelings of boredom, loneliness, disconnection, loss of meaning, fear, anger, avoidance behaviors, and abnormal emotional reactions in the general population (Brooks et al., 2020; Holmes et al., 2020; Pfefferbaum and North, 2020; Pérez-Mengual et al., 2021). In addition, the effects of business and service closures affect populations such as people with children at school, people living alone, elderly people and caregivers, unemployed people, people with low socioeconomic status, and other vulnerable people, compounding the impact of this crisis at different levels (World Health Organization, 2020; Della Gatta et al., 2021). This situation is obviously more complicated for those in a grieving process, or those directly confronting the new virus, such as the healthcare professionals (Braquehais et al., 2020; Murphy and Moret-Tatay, 2021).

While health workers have been recognized for their work during the outbreak, they have also been victims of social stigmatization, work overloads, and lack of material resources, as well as witnessing how their colleagues have been infected or even died (Ruiz-Frutos and Gómez-Salgado, 2021). Furthermore, they might have experienced mental health problems as the general population did, such as stress, anxiety, depressive symptoms, insomnia, denial, anger, and fear (Kontoangelos et al., 2020). It has been pointed out that distress might cause long-term effects on the well-being of professionals (Kang et al., 2020). The size of these problems might also affect attention, perception, and decision-making, processes related to the ability to act against the COVID-19. This issue is of interest when an extreme focus of attention might occur to avoid being infected (Coulthard, 2020). Furthermore, this point seems to be a concern regarding specific variables underlying obsessive-compulsive disorder (OCD).

An individual with symptoms related to OCD experiences an exaggerated preoccupation with danger, hygiene, and/or harm. This response consumes many cognitive resources, generating great distress and associated anxiety, as well as increasing obsessions. In Pauls et al. (2014) model, the person may experience compulsions, or better to say, actions to try to neutralize the distress and anxiety, which provide temporary relief. In other words, anxiety and/or stress can be considered precursors to obsessions and, all these variables predictors to compulsions. Banerjee (2020) claimed that the increased demand for handwashing, the importance of “proper” handwashing, and the need for increasing general hygienic measures to reduce the virus transmission on surfaces, are some of the prevalent factors that could lead to an increase of symptoms. As indicated by Mrklas et al. (2020), assessing the impact of COVID-19 on OCD in healthcare workers and general community might help to mitigate the health footprint. It should be also noted that the information on the distress of healthcare workers and their obsessive-compulsive symptoms in comparison to the general population over time is of interest, as the first group has been reported to pose 12 times more risk for a positive COVID-19 test (Nguyen et al., 2020).

With regard to the healthcare workers, we can find a remarkable profile such as the psychologist. While much has been said about the impact of COVID-19 on healthcare workers who are directly involved in the physical emergency, to our knowledge the literature is rather limited to the impact of COVID-19 in psychologists, who are in charge of the mental health of the community. Thus, the current study aimed to examine the differences in symptomatology associated after the first wave of the COVID-19 outbreak in Spain, across psychologists, healthcare professionals, and the general community. In addition, this study also aimed to test a model of the relationship between mental health and OCD across the groups, as described in previous literature.

One should bear in mind that previous literature found increased levels of stress, anxiety, depressive symptoms, insomnia, denial, anger, and fear in healthcare workers in the COVID-19 pandemic (Barzilay et al., 2020; Kontoangelos et al., 2020). However, it could be expected that the profile of psychologists would be different from other healthcare professionals. In this way, psychologists might employ more self-regulation strategies due to their background and previous training than other professionals (Cleary, 2009). In addition, psychologists might carry out most of their assessments online easily, while most other healthcare professionals need face-to-face appointments to address the physical assessments.

## Method

### Participants

The criteria for inclusion in the sample required participants to be legal adults and residents of Spain. Minor participants, people who did not answer all the questions, and individuals who did not agree to sign the consent to participate were excluded from the current study. As this study involved the use of data from human participants, the project was approved by the Ethical Committee of the *Colegio Oficial de Psicólogos de la Comunidad Valenciana* (COPCV, cert. num. 5AFFD10027A6D61BC585C21E214DB0AE).

A total of 1,928 participants volunteered to participate in the current study. After removing cases with incomplete or duplicated data, a final sample of 1,769 was selected, where 28.9% were men and 71.1% were women. The average age of the participants was 43.21 years (SD = 10.98), with an age range of 18–77 years. For the purpose of the study, even if the psychologist profession is considered as a healthcare one in Spain, participants were divided according to three different groups: general community (*n* = 458), healthcare workers (*n* = 898), and psychologists (*n* = 413). Table 1 depicts the education level, as well as other descriptive data across groups.

**Table 1:** Descriptive data across groups under study.

	**Age (*SD*)**	**Women (%)**	**Education**			**Occupation**		
			**Bachelor**	**Post-graduate**	**Full-time**	**Part-time**	**Unemployed**	**Retired**
General community (25.9%)	47.44 (10.73)	54.60	52.20	47.80	88.40	3.30	0.6	2.30
Healthcare Workers (50.8%)	41.13 (10.74)	71.90	66.80	33.20	78.40	17.50	3.90	0.20
Psychologist (23.3%)	43.06 (10.48)	87.40	24.00	76	74.30	19.40	5.30	1

*SD, standard deviation*.

With regard to the sample of the general community, outside the healthcare environment, this group involved a wide variety of professions related to technical, administrative (6.1%), legal (55%), education (7.6%), management (11.8%), economics (4.6%), consultancy (0.2%), and advisory work (14.7%). On the other hand, the group of healthcare professionals consisted mainly of dieticians (0.1%), veterinarians (25.6%), pharmacist (28%), nurses (4.1%), physiotherapists (28.3%), general practitioners (0.4%), dentists (0.1%), and optometrists (13.4%).

### Instruments

Before administering the questionnaires, a series of sociodemographic questions and data of interest to this study were collected. Specifically, the age, gender, education level, profession, and employment status were requested. Once completed, different measures of psychopathology were administered. The instruments used were the DASS-21 (Lovibond and Lovibond, 1995), and the Y-BOCS (Goodman et al., 1991). These were displayed in the same order for all the participants.

The DASS-21 was chosen because it is an instrument created to screen for depression, anxiety with the scores in the range of 0–63, and stress was measured using a 21-question Likert scale with scoring from 0 to 3. The Spanish version (Daza et al., 2002) has proven to be a reliable tool, providing severity parameters for those constructs in different populations (Ruiz et al., 2017). Also, Wardenaar et al. (2018) found good psychometric properties when the test was Internet-administered. It also has good internal consistency for each of the subscales: Cronbach's α = 0.868 for the Depression subscale (0.878 for the general community, 0.867 for healthcare workers, and 0.846 for psychologist), Cronbach's α = 0.764 for Anxiety (0.793 for the general community, 0.75 for healthcare workers, and 0.731 for psychologist) and Cronbach's α = 0.857 for the Stress subscale (0.867 for the general community, 0.853 for healthcare workers, and 0.846 for psychologist). The cut-off points for depression, anxiety, and stress employed were 5, 4, and 8 for mild symptomatology, respectively, to 14, 10, and 17 for extremely severe symptomatology, as described in Spanish and Colombian populations (Ruiz et al., 2017). The higher scores imply greater psychopathology. This means that a total score of 41, which is relatively far from the maximum score, already suggests high severity symptoms.

The Y-BOCS is a tool designed to screen for obsessions and compulsions through 10 items rated on a 5-point Likert scale. Scores for each item range from 0 to 4. Therefore, greater values imply worse well-being, with a maximum total score of 40. Its translation developed in Peru (Yacila et al., 2016) has shownd good psychometric properties. Moreover, it gives interpretable severity scores described as follows; from 0 to 7, it is assumed as non-clinical manifestations, from 8 to 15, it is considered as mild symptomatology, 16–23 moderate symptoms, 24–31 severe, and 32 up to its maximum as extreme severity. The general internal consistency was optimal with a Cronbach's α = 0.929 in the study. For the subscales, the internal consistency was α = 0.904 in Obsessions (0.894 for the general community, 0.90 for healthcare workers, and 0.902 for psychologists) and α = 0.883 in Compulsions (0.875 for the general community, 0.882 for healthcare workers, and 0.869 for psychologists).

### Procedure

Data were collected through an online survey from the COPCV, following the recommendations made by local institutions to ensure the safety and well-being of every participant regarding COVID-19. Participation in this research was voluntary and completely anonymous, with no incentives for completing the questionnaire. The recruitment was considered a snowball sampling, as an email was sent *via* social media platforms and the COPCV, inviting to share it. At the beginning of the web-based survey, informed consent information was displayed and therefore accepted by every participant. If this consent was not accepted, the process did not continue. The information provided by the participants was completely anonymous, where neither the names nor the IP addresses of the participants were recorded so that they cannot be traced in any case. A pseudonym was requested on a voluntary basis to avoid the duplication of data. This allowed the authors to compare whether two very similar responses submitted consecutively temporarily could be from the same user. In addition, with the pseudonym and the date of submission, participants have the option to withdraw the use of their data later. The questionnaire was available online from July to September 2020.

### Data Analysis

The data were analyzed using the statistical software SPSS IBM for Windows version 23.0, JASP 0.14.1.0 [Computer software], and AMOS 18. Even if the variables were not normally distributed according to Shapiro Wilks, no univariate or multivariate outliers were found. First, a descriptive analysis was carried out, as well as zero-order correlations. As normality and variance homogeneity was not reached, a non-parametric approach was carried out: the Mann–Whitney's *U*-test, as well as the Dunn's multiple comparison test. Structural equation modeling (SEM) was employed to assess the relationship between variables under study across groups using the maximum likelihood method (MLM). Even if SEM does not assume normality but maximum likelihood (ML) does, the asymptotically distribution-free (ADF) technique was also employed. Some of the most widespread goodness-of-fit indices selected for this study are as listed as follows: (i) the χ^2^ goodness-of-fit statistic, (ii) the comparative fit index (CFI), (iii) the Tucker–Lewis index (TLI), and (iv) the root mean square error of approximation (RMSEA).

Finally, a multigroup or invariance analysis was carried out to determine any significant difference in the structural parameters between groups. A hierarchical procedure must be carried out, beginning with an unconstrained one, and adding constraints successively. The logic of this procedure is to test the factorial homogeneity structure across groups, from a stage where all the parameters do not need to be equal to a stage where they must be. In this case, three models were carried out across groups, which tested configural, metric, and scalar invariance.

## Results

Table 2 depicts the descriptive analysis as well as Pearson's coefficients for each group. Regarding the DASS-21 cut-offs, the general community had a high percentage of no manifestations in depression (64.8%), anxiety (70.1%), and stress (58.5%), a low frequency for mild symptoms in depression (12%), anxiety (6.3%), and stress (12.7%), as well as a low one for moderate cut-off in depression (13.8%), anxiety (15.1%), and stress (16.8%), and even lower for extremely severe symptoms (2.8% for depression, 4.1% for anxiety, and 3.3% for stress). On the other hand, the percentage of cases regarding cut-offs in the healthcare workers (not including psychologists) was high for no manifestations in depression (58.9%), anxiety (60.7%), and stress (51.8%). These were lower for mild symptoms in depression (14%), anxiety (8.4%), and stress (14.6%), as well as lower in moderate symptoms for the same subscales described as follows: 18.3, 18.7, and 17.9%, respectively. Severe symptoms depicted even lower percentages following the previous order (5.6, 6.3, and 13.5%) and even lower for extremely severe symptomatology (3.2, 5.9, and 2.2% of the sample). Finally, psychologists had no manifestations for depression (72.6%), anxiety (76.8%), and stress (64.9%), and their mild symptoms also depicted lower percentages (14, 6.3, and 13.6%, respectively). Moderate symptoms were similar to the previous values (9.4, 13.1, and 15.7%, respectively), severe symptom values were lower (2.4, 2.2, and 5.1, respectively), and extremely severe symptom values were even lower (1.5, 1.7, and 0.7%, respectively).

**Table 2:** Descriptive statistics on the variables under study and zero-order correlations among each other.

		**Mean**	***SD***	**Skewness**	**Kurtosis**	**1**	**2**	**3**	**4**	**5**	**6**	**7**
General community	Anxiety. (1)	2.63	2.96	1.26	0.93	1	–	–	–	–	–	–
(*n* = 458)	Depression. (2)	4.05	3.92	1.13	0.73	0.617^**^	1	–	–	–	–	–
	Stress. (3)	6.99	4.44	0.55	−0.26	0.697^**^	0.670^**^	1	–	–	–	–
	Obessions. (4)	3.53	3.41	0.72	−0.47	0.519^**^	0.576^**^	0.503^**^	1	–	–	–
	Compulsions. (5)	2.38	3.04	1.12	0.10	0.425^**^	0.493^**^	0.426^**^	0.772^**^	1	–	–
	Total DASS-21 (6)	13.66	9.99	0.81	0.01	0.848^**^	0.873^**^	0.914^**^	0.603^**^	0.508^**^	1	–
	Total Y-BOCS (7)	5.92	6.07	−0.36	0.11	0.504^**^	0.570^**^	0.496^**^	0.948^**^	0.934^**^	0.593^**^	1
Healthcare worker	Anxiety. (1)	3.39	3.17	0.98	0.29	1	–	–	–	–	–	–
(*n* = 898)	Depression. (2)	4.33	3.98	0.94	0.25	0.620^**^	1	–	–	–	–	–
	Stress. (3)	7.66	4.36	0.26	−0.55	0.702^**^	0.692^**^	1	–	–	–	–
	Obessions. (4)	4.01	3.67	0.56	−0.83	0.523^**^	0.576^**^	0.542^**^	1	–	–	–
	Compulsions. (5)	2.47	3.10	1.07	−0.06	0.439^**^	0.465^**^	0.435^**^	0.764^**^	1	–	–
	Total DASS-21 (6)	15.38	10.19	0.61	−0.24	0.853^**^	0.879^**^	0.916^**^	0.619^**^	0.504^**^	1	–
	Total Y-BOCS (7)	6.48	6.35	0.78	−0.45	0.516^**^	0.559^**^	0.525^**^	0.949^**^	0.928^**^	0.603^**^	1
Psychologist	Anxiety. (1)	2.09	2.51	1.45	1.90	1	–	–	–	–	–	–
(*n* = 413)	Depression. (2)	3.05	3.18	1.45	2.20	0.528^**^	1	–	–	–	–	–
	Stress. (3)	6.12	4.02	0.35	−0.46	0.670^**^	0.609^**^	1	–	–	–	–
	Obessions. (4)	2.65	3.04	1.02	0.18	0.556^**^	0.454^**^	0.538^**^	1	–	–	–
	Compulsions. (5)	1.09	2.13	2.11	3.57	0.411^**^	0.316^**^	0.351^**^	0.617^**^	1	–	–
	Total DASS-21 (6)	11.26	8.37	0.83	0.38	0.822^**^	0.831^**^	0.912^**^	0.597^**^	0.412^**^	1	–
	Total Y-BOCS (7)	3.73	4.65	1.35	1.23	0.550^**^	0.440^**^	0.511^**^	0.933^**^	0.858^**^	0.577^**^	1

*SD, standard deviation*.

^*^p < 0.05.

** *p < 0.01*.

The severity scores of the Yale–Brown Obsessive Compulsive Scale in terms of cut-off were described as follows: (a) no manifestations of OCD were found for 66.8% of the general community; 62.5% of healthcare workers; and 80.6% of psychologists; (b) mild OCD symptoms for 22.9, 25.9, and 17.4%, respectively; (c) moderate OCD symptoms for 10, 11, and 1.9%, respectively; (d) severe OCD symptoms for 0.2% of the general community and 0.6% of the healthcare workers. No severe symptoms were found for the group of psychologists.

Overall higher scores were found for the healthcare workers and lower scores for psychologists. Strong correlations were found across mental health, in terms of anxiety, depression, and stress, and Y-BOCS subscales for all the groups. As variance homogeneity was not reached across the groups through Levene's test, a non-parametric approach was chosen. In this way, the Kruskal-Wallis test showed that there was a statistically significant difference in the Anxiety scores between professional groups [χ^2^(2) = 63.47, *p* < 0.001], Depression [χ^2^(2) = 27.73, *p* < 0.001], Stress [χ^2^(2) = 35.15, *p* < 0.001], Compulsions [χ^2^(2) = 80.23, *p* < 0.001], and Obsessions [χ^2^(2) = 41.10, *p* < 0.001].

**Figure 2:**
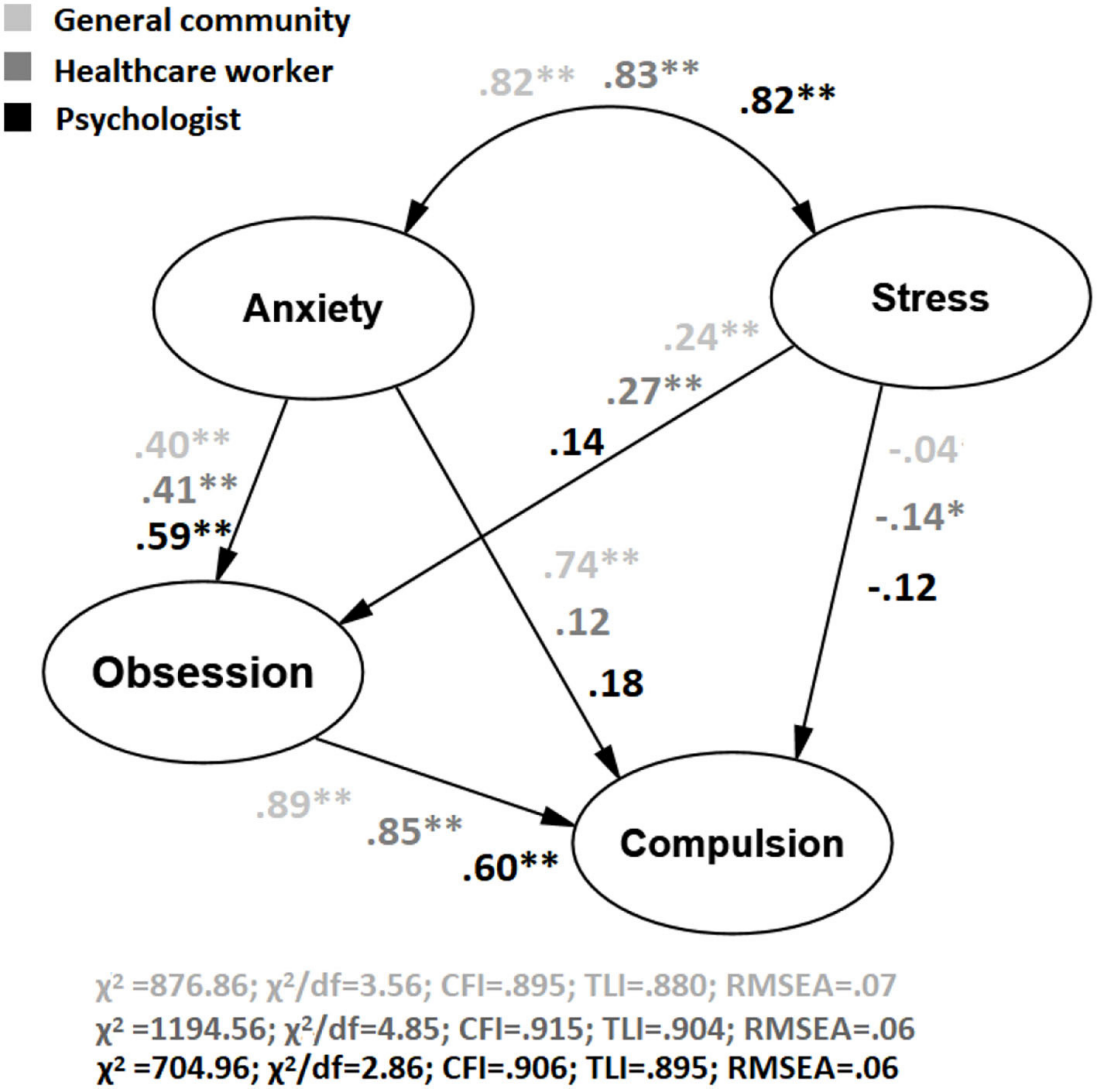
Structural equation model testing an adapted model from Pauls et al. (2014) on Anxiety, Depression, and OCD across groups under study between the first wave and the second wave in Spain. * *p* < 0.05, **p < 0.01.

Dunn's *post-hoc* was also carried out. This test provides its own *p*-value, as does Bonferroni's test and the Holm correction. As can be seen in Table A1, the group scores are significantly independent except for some comparisons between the health workers group and the general community (Depression and Compulsions). To reach the second aim of the study, we tested the goodness of fit of an SEM on each group through MLM after reviewing the normality of the residuals (as shown in Figure 2). The Depression subscale from DASS-21 was not included in this model, as previous approaches in OCD had focused on anxiety and stress. Figure 2 illustrates that the model obtains an acceptable fit for the three groups.

An ADF method was also employed, as previous assumptions on data normality were not reached. This analysis depicted an adequate goodness of fit for the psychologist group (χ^2^/df = 3.26; CFI = 0.914; TLI = 0.915; RMSEA = 0.07), and almost adequate for the general community (χ^2^/df = 4.01; CFI = 0.78; TLI = 0.78; RMSEA = 0.08), and the healthcare workers (χ^2^/df = 3.30; CFI = 0.77; TLI = 0.77; RMSEA = 0.05). Finally, a multigroup analysis was carried out to test invariance in the model. As depicted in Table A2 χ^2^/df and CFI goodness of fit was poor on model 3 in comparison with the previous model.

## Discussion

Since the beginning of the COVID-19 outbreak, the literature has assessed the psychological impact derived from this pandemic. In this way, higher levels of emotional symptoms have been found in the general community, such as greater worries, stress, anxiety, depression, among others (Sandín et al., 2020; Biondi et al., 2021; Murphy et al., 2021). Moreover, higher levels of stress, anxiety, and depression were found in the healthcare workers (Dosil et al., 2020). According to Hao et al. (2020), an increase in anxiety levels is mainly linked to uncertainty regarding the management of the emergency.

China has been a point of reference in the literature for being the first country to tackle the virus. The previous literature in the first wave (Choi et al., 2020), observed that ~25% of the population in China had high levels of both anxiety and depression. Similar results were described by Wang et al. (2020), who evaluated 1,210 participants from almost 200 cities in China using the DASS-21 questionnaire, finding that 53.8% of those surveyed rated the psychological impact of the outbreak as moderate or severe. Last, similar results for healthcare workers have been found all over the world, e.g., Switzerland (Weilenmann et al., 2021) or Italy (Portoghese et al., 2021), among others. After the first wave in Spain, the current results also suggest higher levels of distress among healthcare workers, while psychologists seem to be more resistant to this situation. Moreover, healthcare workers did not statistically differ from the general community in depression and obsessions.

Ettman et al. (2020) found that the levels of depression for adults in the United States had been tripled in this period, proposing different explanations. One of these is related to the moment in which data were recruited. Of note, the current study was conducted in the summer period, when restrictive measures were relaxed in Spain. This could be understood as a limitation, but we also consider that these results could be of interest for future measures and decisions taken by governments. Of note, participants were not examined according to the severity of their work toward COVID-19, as suggested in previous literature (Hou et al., 2020). Additionally, some information of interest was not recruited, e.g., whether the participants had a previous background (or concurrent diagnosis) of mental or physical health, which can impact the symptoms they are assessing. Therefore, long-term studies that include these variables, might show the effects of psychological burnout over time, are of interest.

It should be noted that psychologists showed lower scores on compulsions and obsessions. As some authors point out, it is important to distinguish the onset of irrational fears from rational behavior (Aardema, 2020; Banerjee, 2020). In this way, it seems that psychologists might have balanced more these practices in their work, in comparison with the general community and healthcare workers. As also noted above, psychologists would not be as exposed as other professionals. However, their scores were also lower than the general community. This is of particular interest for the prevention programs, especially in those groups of healthcare professionals who seem to be so vulnerable. The role of the psychologist in this area seems to be crucial.

Current results seem to support the previous theoretical proposals (Pauls et al., 2014), and the role of background and previous experiences of the professionals in these models, as different relationships between anxiety and stress in obsessions and compulsions have been found. Likewise, it is supported that anxiety is a precursor of obsessions in all the groups, but the effect of stress could be modified across them. This result is of special interest, both for theoretical and applied levels to implement current theoretical models, training, and treatment programs. On the other hand, factor loadings were invariant across populations, offering a model that seems to be invariant to some extent. However, one of the limitations of this study is that the invariance did not reach the scalar level, also known as strong invariance. As Bollen (1989) indicated, this might be indicative of potential measurement bias among items intercept. Therefore, differences in the way of responding and rating the items might occur.

The main limitations of this study can be described as follows: (i) The sample was selected through non-probability sampling, which can introduce distortions in the results; (ii) Biases might occur as data were recruited in a self-report way in a range of 3 months when there were restrictions and cases changed in the space of a few days; (iii) The unbalanced sample depicted a significantly higher number of women than men; (iv) The results are indicative but not a diagnose, they serve to warn about the presence of manifestations related to obsessive and compulsive symptoms, as well as stress and, consequently, to facilitate the decision of whether or not to consult specialists; and (v) Several characteristics of interest regarding the samples under study were not recruited, such as socio-economic condition or direct experience with the virus. Particularly, data related to whether participants were infected or had dealt with loss because of the COVID-19 seems to be crucial. Thus, this is a limitation of the current study, making it difficult to determine the predictive value of being in contact with the virus.

Regarding future lines of research, there are several questions that remain open. One of these lines should be focused on the clinical samples with non-self-reported methods, allowing addressing psychopathological differences (Burrai et al., 2020; Cordellieri et al., 2021). As Cullen et al. (2020) claimed, the study of psychological factors is useful, as these might interfere with the adherence to public health measures, such as vaccinations and the ability of the population to cope with the threat of infection and consequent losses.

## Conclusions

The aim of this study was to examine the distress level associated with mental health across psychologists, healthcare professionals, and the general community, after the first wave of the COVID-19 outbreak in Spain. Moreover, a model on the relationship between mental health and OCD across groups, as described in previous literature, was also examined. This is of interest at a clinical level, because of the unique information that this moment in time offers us on the variables under study and their differences between the groups under study. The main results can be sorted as follows: (i) Healthcare workers and the general community depicted similar results in anxiety and stress, as well as obsessions; (ii) The group of psychologists described better scores than other groups under study; (iii) Stress and anxiety did not predict compulsions in the group of psychologists; (iv) Anxiety predicted obsessions for all the professions, while the relationship of this variable with stress was different for each group; and (v) Invariance reached a full metric level.

The importance of identifying distress levels in the different profiles under study might make it possible to implement current interventions, emphasizing the role of psychologists to mitigate the effects of this pandemic. Although extensive literature studied the pandemic effects on the mental health of healthcare workers, to our knowledge, the studies on the psychologist-specific profile are limited.

## Data Availability Statement

The raw data supporting the conclusions of this article will be made available by the authors, without undue reservation.

## Ethics Statement

The studies involving human participants were reviewed and approved by the Ethical Committee of the Colegio Oficial de Psicólogos de la Comunidad Valenciana (COPCV). The patients/participants provided their written informed consent to participate in this study.

## Author Contributions

All authors listed have made a substantial, direct and intellectual contribution to the work, and approved it for publication.

## Conflict of Interest

The authors declare that the research was conducted in the absence of any commercial or financial relationships that could be construed as a potential conflict of interest.

## Publisher's Note

All claims expressed in this article are solely those of the authors and do not necessarily represent those of their affiliated organizations, or those of the publisher, the editors and the reviewers. Any product that may be evaluated in this article, or claim that may be made by its manufacturer, is not guaranteed or endorsed by the publisher.

## Appendix

**Table A1:** Dunn's *post-hoc* comparisons across groups.

**Comparison**		***Z***	**W_**i**_**	**W _**j**_**	***p***	***p*_**bonf**_**	***p*_**holm**_**
Anxiety	Healthcare-psychologist	7.522	974.498	749.360	<0.001	<0.001	<0.001
	Healthcare-community	4.935	974.498	831.834	<0.001	<0.001	<0.001
	Psychologist-community	−2.414	749.360	831.834	0.008	0.024	0.008
Depression	Healthcare-psychologist	5.252	932.105	773.769	<0.001	<0.001	<0.001
	Healthcare-community	1.345	932.105	892.944	0.089	0.268	0.089
	Psychologist-community	−3.464	773.769	892.944	<0.001	<0.001	<0.001
Stress	Healthcare-psychologist	5.797	949.233	773.608	<0.001	<0.001	<0.001
	Healthcare-community	3.067	949.233	859.505	0.001	0.003	0.002
	Psychologist-community	−2.484	773.608	859.505	0.006	0.019	0.006
Obsessions	Healthcare-psychologist	6.411	945.287	753.724	<0.001	<0.001	<0.001
	Healthcare-community	2.083	945.287	885.172	0.019	0.056	0.019
	Psychologist-community	−3.855	753.724	885.172	<0.001	<0.001	<0.001
Compulsion	Healthcare-psychologist	8.585	944.409	701.630	<0.001	<0.001	<0.001
	Healthcare-community	0.386	944.409	933.871	0.350	1.000	0.350
	Psychologist-community	−7.196	701.630	933.871	<0.001	<0.001	<0.001

*Healthcare for healthcare professionals, and Community for the general community*.

**Table A2:** Goodness-of-fit statistics across groups: a summary.

**Model**	**χ^**2**^**	**Df**	**χ^**2**^/df**	**CFI**	**RMSEA**	**Decision**
Model 1: Configural invariance (baseline model)	2,776.64	728	3.76	0.907	0.040	-
Model 2: Full metric invariance	2,859.91	778	3.67	0.905	0.039	Accept
Model 3: Full metric and scalar invariance	2,924.21	788	3.71	0.903	0.039	Reject
